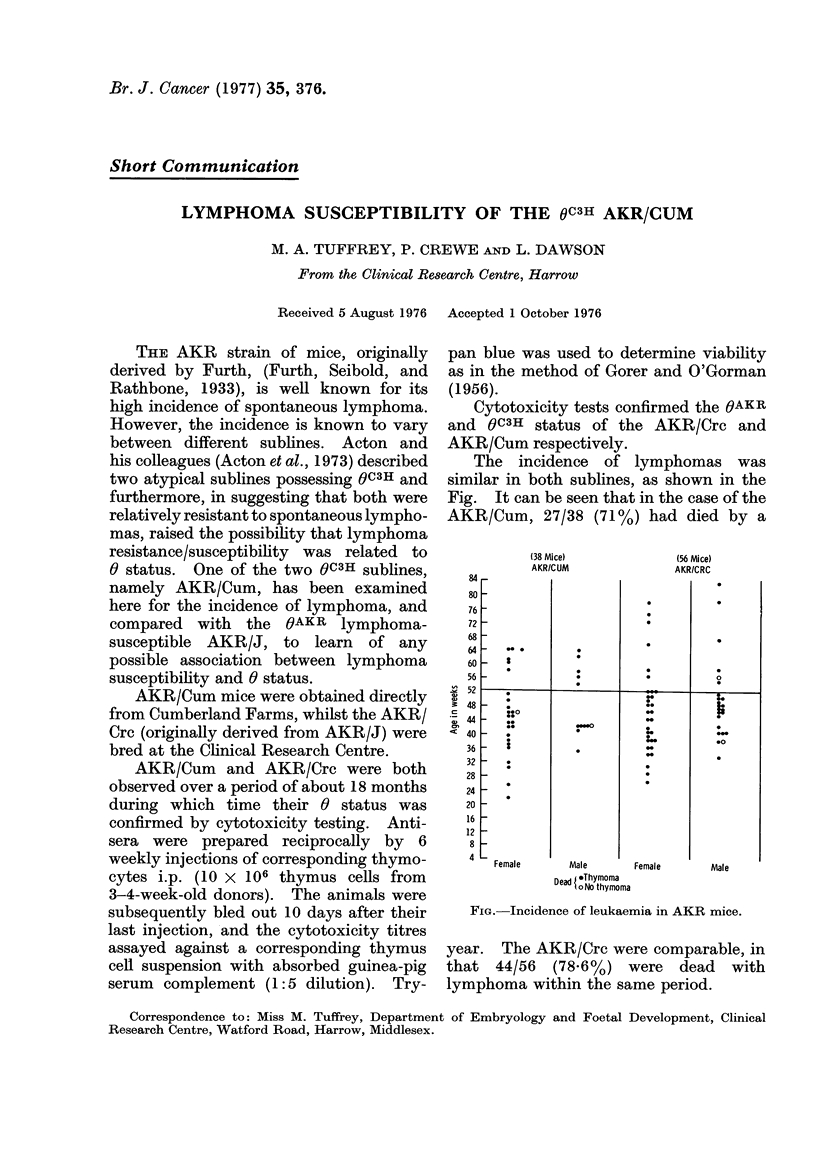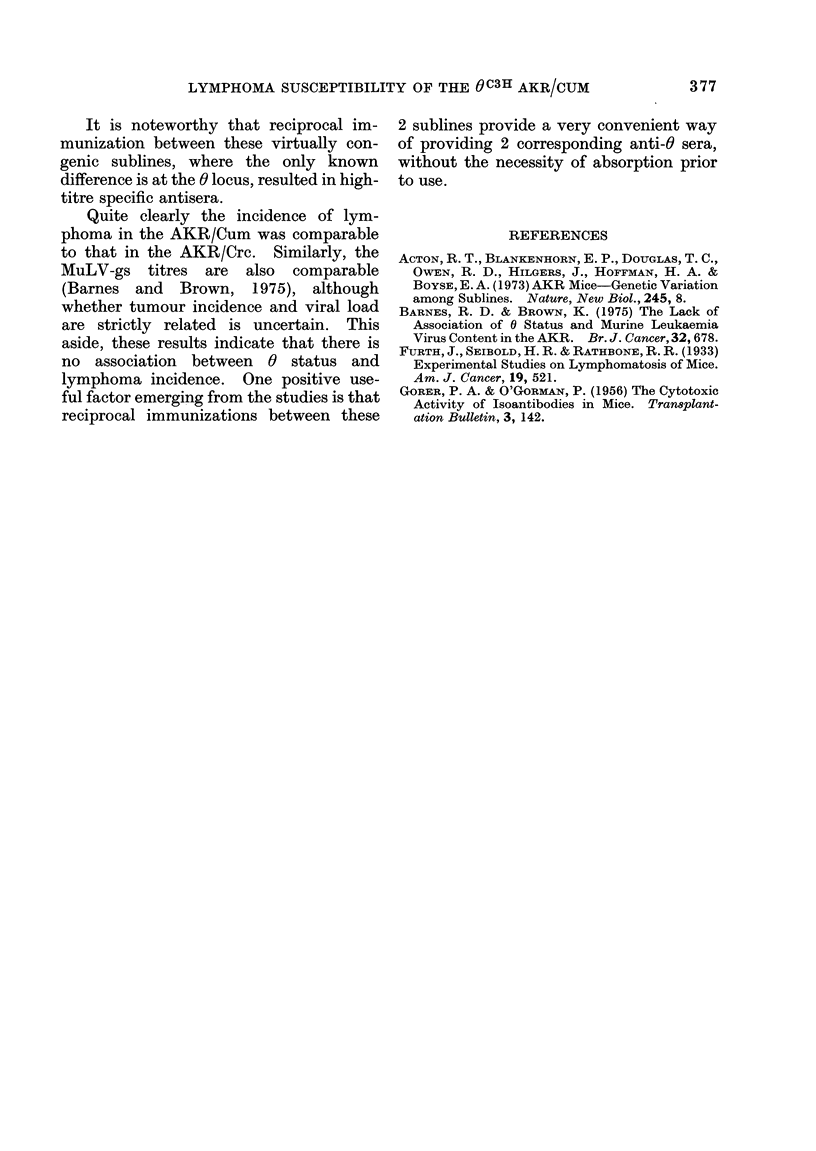# Lymphoma susceptibility of the thetaC3H AKR/CUM.

**DOI:** 10.1038/bjc.1977.53

**Published:** 1977-03

**Authors:** M. A. Tuffrey, P. Crewe, L. Dawson


*Br. J. Cancer* (1977) **35**, 376.

***Short Communication***

### LYMPHOMA SUSCEPTIBILITY OF THE $\theta^{C3H}$ AKR/CUM

M. A. TUFFREY, P. CREWE AND L. DAWSON

*From the Clinical Research Centre, Harrow*

Received 5 August 1976 Accepted 1 October 1976

THE AKR strain of mice, originally derived by Furth, (Furth, Seibold, and Rathbone, 1933), is well known for its high incidence of spontaneous lymphoma. However, the incidence is known to vary between different sublines. Acton and his colleagues (Acton *et al.*, 1973) described two atypical sublines possessing $\theta^{C3H}$ and furthermore, in suggesting that both were relatively resistant to spontaneous lymphomas, raised the possibility that lymphoma resistance/susceptibility was related to $\theta$ status. One of the two $\theta^{C3H}$ sublines, namely AKR/Cum, has been examined here for the incidence of lymphoma, and compared with the $\theta^{AKR}$ lymphoma-susceptible AKR/J, to learn of any possible association between lymphoma susceptibility and $\theta$ status.

AKR/Cum mice were obtained directly from Cumberland Farms, whilst the AKR/Crc (originally derived from AKR/J) were bred at the Clinical Research Centre.

AKR/Cum and AKR/Crc were both observed over a period of about 18 months during which time their $\theta$ status was confirmed by cytotoxicity testing. Antisera were prepared reciprocally by 6 weekly injections of corresponding thymocytes i.p. ($10 \times 10^6$ thymus cells from 3–4-week-old donors). The animals were subsequently bled out 10 days after their last injection, and the cytotoxicity titres assayed against a corresponding thymus cell suspension with absorbed guinea-pig serum complement (1:5 dilution). Trypan blue was used to determine viability as in the method of Gorer and O'Gorman (1956).

Cytotoxicity tests confirmed the $\theta^{AKR}$ and $\theta^{C3H}$ status of the AKR/Crc and AKR/Cum respectively.

The incidence of lymphomas was similar in both sublines, as shown in the Fig. It can be seen that in the case of the AKR/Cum, 27/38 (71%) had died by a year. The AKR/Crc were comparable, in that 44/56 (78·6%) were dead with lymphoma within the same period.

FIG.—Incidence of leukaemia in AKR mice.

Correspondence to: Miss M. Tuffrey, Department of Embryology and Foetal Development, Clinical Research Centre, Watford Road, Harrow, Middlesex.

It is noteworthy that reciprocal immunization between these virtually congenic sublines, where the only known difference is at the $\theta$ locus, resulted in high-titre specific antisera.

Quite clearly the incidence of lymphoma in the AKR/Cum was comparable to that in the AKR/Crc. Similarly, the MuLV-gs titres are also comparable (Barnes and Brown, 1975), although whether tumour incidence and viral load are strictly related is uncertain. This aside, these results indicate that there is no association between $\theta$ status and lymphoma incidence. One positive useful factor emerging from the studies is that reciprocal immunizations between these 2 sublines provide a very convenient way of providing 2 corresponding anti-$\theta$ sera, without the necessity of absorption prior to use.

### REFERENCES

Acton, R. T., Blankenhorn, E. P., Douglas, T. C., Owen, R. D., Hilgers, J., Hoffman, H. A. & Boyse, E. A. (1973) AKR Mice—Genetic Variation among Sublines. *Nature, New Biol.*, **245**, 8.

Barnes, R. D. & Brown, K. (1975) The Lack of Association of $\theta$ Status and Murine Leukaemia Virus Content in the AKR. *Br. J. Cancer*, **32**, 678.

Furth, J., Seibold, H. R. & Rathbone, R. R. (1933) Experimental Studies on Lymphomatosis of Mice. *Am. J. Cancer*, **19**, 521.

Gorer, P. A. & O'Gorman, P. (1956) The Cytotoxic Activity of Isoantibodies in Mice. *Transplantation Bulletin*, **3**, 142.